# Early Phase Psychiatric Response for Children and Adolescents After Mass Trauma: Lessons Learned From the Truck-Ramming Attack in Nice on July 14th, 2016

**DOI:** 10.3389/fpsyt.2019.00065

**Published:** 2019-02-20

**Authors:** Florence Askenazy, Morgane Gindt, Lucie Chauvelin, Michèle Battista, Fabian Guenolé, Susanne Thümmler

**Affiliations:** ^1^University Department of Child and Adolescent Psychiatry, Children's Hospital of Nice CHU-Lenval, Nice, France; ^2^University Côte d'Azur, CoBTek, Nice, France; ^3^University Department of Child and Adolescent Psychiatry, University Hospital of Caen, Caen, France; ^4^INSERM UMR-S 1077, Caen, France

**Keywords:** child and adolescent psychiatry, disaster, terrorism, emergency psychological response, trauma related disorders, CUMP

## Abstract

Recent years have seen a multiplication of terrorist attacks in public places across European and North American countries, thus heightening the need for public mental health planning and response strategies focused on the special needs of children and their families. The present article retrospectively analyzes the early phase psychiatric response for children and adolescents after the truck attack in Nice on July 14th, 2016. In addition, lessons which can be drawn from it will be discussed, with a focus on organizational challenges in the early phase. During the first 2 weeks after the attack, 668 individuals have been registered at the medico-psychological emergency unit of the Children's Hospitals of Nice, including 365 (54.6%) children and adolescents of all ages. Overall, 146 child and adolescent mental health care professionals participated in this specific facility, including 75 psychiatrists and psychologists. The implementation of the medico-psychological emergency unit dedicated to the pediatric population has been an indispensable and unprecedented public health challenge in our country. Future studies are needed in order to evaluate and to improve the efficiency of the individual as well as collective impact of early phase psychiatric interventions dedicated for children and adolescents after mass trauma.

## Introduction

The truck-ramming attack in Nice, France, on the evening of July 14th, 2016 (see Box 1), is one of several terrorist attacks which occurred in public places across European countries in recent years (1). At the time of this violent event more than 30,000 people were present in the area with a large number of babies, children and adolescents (2). This type of attack is categorized as acts of indiscriminate and retributive mass terrorism (3), i.e., directed against a huge number of people as targets of opportunity, without a precise demand or objective but destroying them and in this way generally inspiring terror. It therefore constitutes the highest existing traumatic stressor (4), as it combines malevolent intent, extreme violence and harmfulness against unprepared victims, collective stress, and maximal media coverage in the following period. Moreover, it generally strikes entire families, including children and adolescents, which greatly impacts community resiliency (5, 6). For these reasons, heightened awareness of the need for effective public mental health planning and response strategies focused on the special needs of children and their families are mandatory (7, 8).

Box 1 **The truck-ramming attack, Nice, July 14^th^, 2016**

On Thursday, July 14^th^, 2016, at 10:33 p.m. in Nice, a gunman conducting a heavy truck rushed quickly at the crowd along the seafront boulevard (the Promenade des Anglais), killing 86 people, including ten children, and injuring about 500 other people. This attack occurred during the summer holiday period, on the evening of the national day, just a few minutes after the traditional fireworks. About 30.000 people were at the scene, including many children and their families. People on the promenade heard and saw the truck rushing at them, running over others and projecting bodies. They ran away in all directions, sometimes jumping with their children on the pebble beach several meters below. A huge panic stampede also spread out along the truck's trail, with people running among casualties toward adjacent streets; some children lost sight with their parents in the crush. Other people witnessed the whole scene from windows and balconies of apartments and hotel rooms along the seafront. The ramming truck stopped at 10:50 p.m., after a course of 1.7 kilometers through the crowd, and the driver was killed by policemen. Some people around witnessed the gunfight, the rest of the dense crowd in the area only heard it and the following police sirens, without knowing what was actually happening. Most of people at the scene stayed confined for several hours in building hallways, restaurants or shops, where they received on social networks some alarming fake news about multiple attacks across the town. Going back to their home or vehicle, a number of families had to walk again along the Promenade des Anglais, and were thus exposed to the bloody bodies of victims.

The essentials of early medico-psychological intervention after mass trauma include two distinct yet simultaneous main objectives. The first one is to reduce vulnerability to stress in children and families, mainly by promoting a sense of safety and calming (9), in order to prevent chronic stress. The second one is to identify children and adolescents who are at high risk for subsequent psychopathology according to their individual and family antecedents and current clinical status (4, 10, 11), and to refer them for follow-up psychiatric interventions. Later and persistent symptoms and disorders include mainly post-traumatic stress disorder (PTSD), but also other mental disorders like anxiety, depression or attention deficit hyperactivity (ADHD) disorders (12, 13), as well as various developmental symptoms in younger children (14). Achieving these two objectives in the context of an early phase disaster response raises many organizational challenges (15), and the recent multiplication of mass terrorist attacks thus reinforces the necessity that child and adolescent mental health professionals contribute to preparedness of health systems to address such events.

Only two guidelines provide recommendations about stress reactions in emergency situations for children and adolescent: (1) the NICE guidelines (16) and (2) the Phoenix guidelines (17). They underline trauma-related symptoms in the pediatric population such as sleep disturbances, the appearance of new fears as well as developmental regression. Nevertheless, both guidelines are not specific for terrorist attacks.

As compiling and reviewing mental health data from past attacks is the first way to optimize and enhance preparedness of health systems to mass terrorism, the purpose of this article was to retrospectively analyze emergency psychiatric response for child and adolescents after the July 14th, 2016 terrorist attack in Nice, and to discuss lessons which can be drawn from it, with a focus on organizational challenges in the early phase.

The facility has been located in the Children's Hospitals of Nice which comprises all public pediatric services in the greater Nice area. Its activities are located near the center of the city of Nice on the seafront boulevard -the *Promenade des Anglais*—at 200 m from the beginning of the attack. This experience has been an unprecedented public health challenge because of three mean aspects. First, the facility was specifically dedicated to the pediatric population and their families; second, it had to face a large number of survivors in a short lap of time; and third, it therefore had to organize the intervention of an important number of health professionals.

## Methods

A retrospective analysis was performed of the medico-psychological response of the first two weeks at the Children's Hospitals of Nice CHU-Lenval (*Hôpitaux Pédiatriques de Nice CHU-Lenval*) to the July 14th, 2016 attack.

Qualitative and quantitative data were collected for the period of July 14–28th, 2016 from: real-time and time-stamped (1) hospital admission records, (2) computerized and handwritten logs, (3) handover protocols, and (4) from patients' charts and *ORSAN*-forms (a standardized disaster plan medical record; see Box 2.1) for a review. Cross-checking interviews of key informants (on-scene team members) completed data collection when necessary.

Box 2 **Definitions of French emergency and child and adolescent psychiatry systems**

**1**. ***ORSAN*** **plan** (*plan ORSAN*): the nationwide French emergency plan to face the sudden healthcare needs due to an accident, a disaster, an epidemic or a serious climatic event. *ORSAN* stands for *Organisation de la Réponse du système de SANté en situations sanitaires exceptionnelles* (organization of the health system response in exceptional healthcare situations). Defined in 2014 under its current form, the *ORSAN* plan had been activated two times before the Nice attack: during the influenza epidemic of winter 2014–2015, and during the November 13th, 2015 terrorist attacks in Paris. When the *ORSAN* plan is activated, medical information about each patient rescued is recorded on a standardized form (*ORSAN* form). It comprises personal data (name, age, address, phone number, email), informations about the traumatic event (date, type, implication, duration) as well as clinical evaluation (symptoms, severity).

**2. White plan** (*plan blanc*): the local emergency plan designed by each hospital center as part of the *ORSAN* plan. An hospital's white plan principally defines emergency procedures for rescue and coordination of care resources in case of a massive influx of patients, and includes a psychiatric component.

**3. Medico-psychological emergency cell** (*cellule d'urgence médico-psychologique, CUMP*): the mental healthcare mobile unit which provides immediate and short-term intervention for collective psychological trauma in each French department (18). It includes psychiatrists, psychologists and nurses, and aims at treating the disturbing symptoms of stress, ensuring the triage and short- to mid-term referral of patients at psychiatric risk, and informing about acute stress symptoms and their evolution. Medico-psychological emergency cells were created in 1997 following the 1995 subway bombing in Paris. They constitute a French healthcare specificity. No specific pediatric component was included in formation of medico-psychological emergency cells, which has been recognized as a need in the recent years, particularly following the Nice attack (8).

**4. Inter-sector of infant-juvenile psychiatry** (*intersecteur de psychiatrie infanto-juvénile*): the local public department of child and adolescent psychiatry. An inter-sector of infant-juvenile psychiatry entails a pluri-professional team, and aims at providing outpatient, inpatient, and intermediate (day hospital) specialized treatments in child and adolescent psychiatry over an area corresponding to approximately 180 000 residents. The French territory is divided into 320 inter-sectors of infant-juvenile psychiatry.

**5. Healthcare emergencies preparation and response team** (*Equipe de Préparation et de Réponse Aux Urgences Sanitaires, EPRUS*): the French public agency designed for helping state officials and health operators responding to exceptional healthcare situations in France and abroad. It handles operationality of 2000 mobilizable health reserves volunteers, and strategic stocks of health products.

Collected data concerned clinical consultations (age, sex, date), the medico-psychological emergency cell organization (number and type of professionals) as well as group debriefing (type, date). We employed descriptive statistics using mean and percentages.

The data collection has been performed in accordance with the ethical standards of the 1964 Declaration of Helsinki and its later amendments, and has been declared to the French national commission on informatics and liberty (CNIL n° 2181236).

## Results

**Figure 1:**
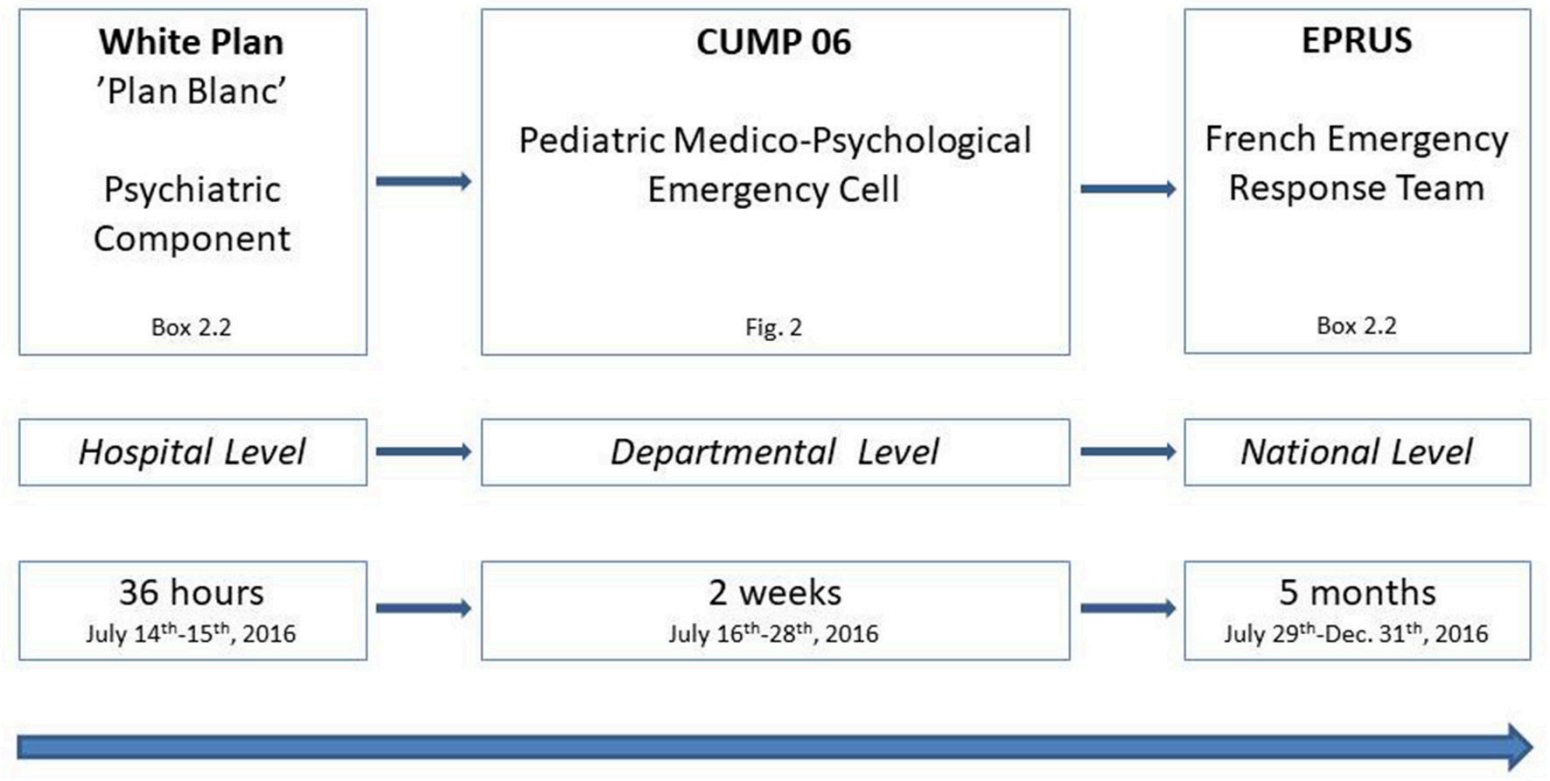
Organization of Specific Psychological Care for children and adolescents after the Nice terrorist attack of July 14th, 2016. CUMP, Cellule d'Urgence Médico-Psychologique; EPRUS, Equipe de Préparation et de Réponse Aux Urgences Sanitaires.

Because of its proximity to the attack site, injured children and adults began arriving independently at the Children's Hospitals just a few minutes after the attack on July 14th, 2016, thus without the benefit of pre-hospital triage. This was the case for 30 of the 44 patients (32 children and 12 adults) admitted at the emergency ward during the following hour (19). This caused an initial disorganization of regular functioning, until the ORSAN plan (see Box 2.1) and the “white plan” of the Children's Hospitals of Nice CHU-Lenval (see Box 2.2, Figure 1) were activated (23:30 p.m.), leading to a change in organization of patient flow and to staff and technical reinforcements. Among patients admitted immediately after the attack, 13 were in critical condition, of whom 7 needed immediate intensive care and/or surgery and 6 deceased during first aid (19).

As in all French departments, the local medico-psychological emergency cell of the department *Alpes-Maritimes* (see Box 2.3, Figure 1) displayed no specific pediatric component before this event (8). Thus, the psychiatric component of the Children's Hospitals “white plan” was the only unit immediately operational for child and adolescent psychological interventions in collective psychological trauma in the greater Nice area. It included 5 child psychiatrists, 1 psychologist, 3 nurses, 1 secretary and 2 health executives. This team was fully operational 1 hour after activation of the “white plan.” In addition, psychiatric interventions at the emergency ward were carried out by a child and adolescent psychiatrist, psychologist and the junior psychiatrist on duty this night.

Logistical and organizational aspects were handled primarily by the health executives (20). A calm area outside the emergency ward was implemented for the medico-psychological unit, including a reception specifically dedicated to the children and their families, a waiting space, and four consulting rooms. People in need of psychological interventions were accompanied there from the emergency ward by the psychologist. In the waiting place, persons could keep hydrated, take food, and have covers at their disposal. Some distressed people were in search of a missing child or adult, so that the health executive had to handle searches. The initial disorganization of administrative procedures did not allow an exhaustive account of psychiatric interventions during the night; particularly, the numerous ones carried out directly at the emergency ward could not be recorded (2). It can be mentioned however that two adult patients manifested severe dissociative symptoms and thus required transfer to the adult psychiatric emergency department in another hospital center [Pasteur Hospital; (21)]. Seventeen patients were registered at the medico-psychological unit, 5 children and 12 adults; all displaying acute stress reaction. Mental health professionals were also asked to contribute announcing death of a family member or mutilating surgery in some cases, and to bring emotional support to some of their colleagues of the emergency staff.

**Figure 2:**
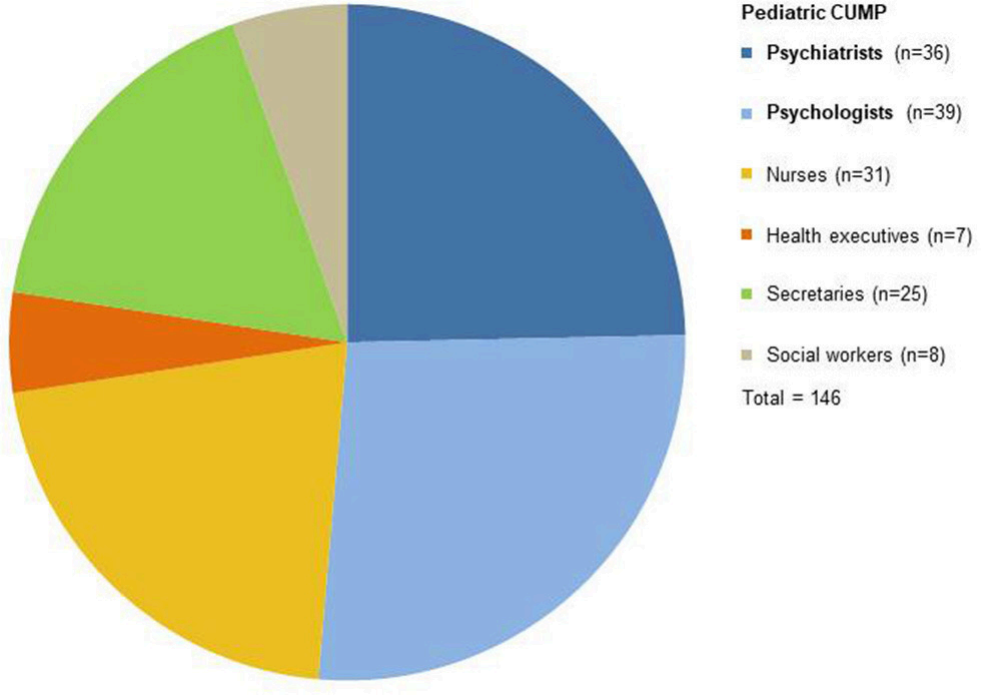
Mental health professionals participating in the Pediatric Medico-Psychological Emergency Cell (CUMP) accross the period of July 15–28th, 2016.

This “white plan” medico-psychological emergency unit was renewed the following day (July 15th, 2017), and soon instituted by national health authorities as a post-attack pediatric medico-psychological emergency cell, attached to the medico-psychological emergency cell of the French department *Alpes-Maritimes* (*Cellule d'Urgence Médico-Psychologique, CUMP 06*, Figure 1). Staff schedules were organized for the following days, allowing two shifts, with a 1-h overlap at midday for information handover (9:00 a.m.−5:30 p.m. and 4:30 p.m.−11:00 p.m.). From July, 14–28th, 2016, 26 child and adolescent psychiatrists, 32 psychologists, 28 nurses, 8 social workers, 25 secretaries, and 6 health executives participated part time to the staff; most of them were members of the of the three child and adolescent psychiatry departments of the Children's Hospitals of Nice CHU-Lenval, some psychiatrists and psychologists were in private practice. Mean numbers of working days at the unit during the period was as follow: psychiatrists = 1.8; psychologists = 1.8; nurses = 1.6; social workers = 2.0; secretaries = 1.5; and health executives = 5.7. From July 20–28th, mental health professionals from regional departments of child and adolescent psychiatry and from four French medico-psychological emergency cells progressively reinforced the staff, bringing its workforce to a total number of 146 professionals for the whole period, including 36 psychiatrists, 39 psychologists, 31 nurses, 8 social workers, 25 secretaries, and 7 health executives (Figure 2).

The primary clinical principle of the unit was to promote a relative safety and calming, through providing a secure and well-treating place, and meeting of safe persons. Ideally, the child/adolescent and his/her family were received by a professional duo, including a child and adolescent psychiatrist, and children aged under 6 years and their family were received by a professional trio (so that a nurse could stay apart with the child when it proved necessary to see parents separately). However, these conditions were not always feasible, and part of consultations (37.9%) was made by single professionals. These clinical consultations, usually long ones lasting sometimes more than 1 h, entailed strong emotional expression. In particular, parents frequently expressed distressing feelings of guilt and shame regarding the facts of having taken their children to the place where the attack happened, and/or of not having managed protecting them.

Consultations in themselves had several specific aims. First, they aimed at providing emotional support and favoring positive emotions and support within the family (9). They also helped to restore temporal chronology before and after the event. Information was also given in order to restructure irrational fears, including about the normality of acute stress reaction and its expected course during the following weeks. Recommendations were given to limit exposure to news media and the amount of talking about the trauma for most anxious children, together with warning about over-protectiveness. Maintenance of planned activities if possible was advised (including going on holidays, to the day nursery, childminder, or local holiday activity center for schoolchildren). No critical incident stress debriefing or related intrusive debriefing techniques were used. Parents of children and adolescents with mild to moderate acute stress reaction were advised to call a free-toll phone number—specifically implemented for helping and orienting victims of the attack and advertised in mass media—if symptoms of acute stress reaction exacerbated or lasted more than 1 month. Another aim of consultations was to identify children and adolescents who displayed extreme or complicated stress reactions, or acute stress reaction intertwined with prior mental disorders, and thus needed follow-up care within the unit, or referral to the inter-sector of infant-juvenile psychiatry (*intersecteur de psychiatrie infanto-juvénile*; Box 2.4). Referral was facilitated by the presence within the team of health professionals and social workers from several inter-sectors. When clinically necessary, adults also were guided toward clinical departments of adult psychiatry, in particular the adult medico-psychological emergency cell, implemented at the Pasteur Hospital of the University Hospitals of Nice (CHU) a few kilometers away (21). In order to facilitate information handover between both units, a bi-daily meeting was organized between the medical coordinators of the adult and the child and adolescent medico-psychological emergency cells during the week after the attack, which turned into a phone conference during the following week.

In addition, the medical coordinator assured the exchange with the national CUMP, other medical departments (e.g., pediatric departments, adult psychiatry, legal medicine), the different administrations (e.g., regional prefecture), victim's organizations, justice as well as media.

**Figure 3:**
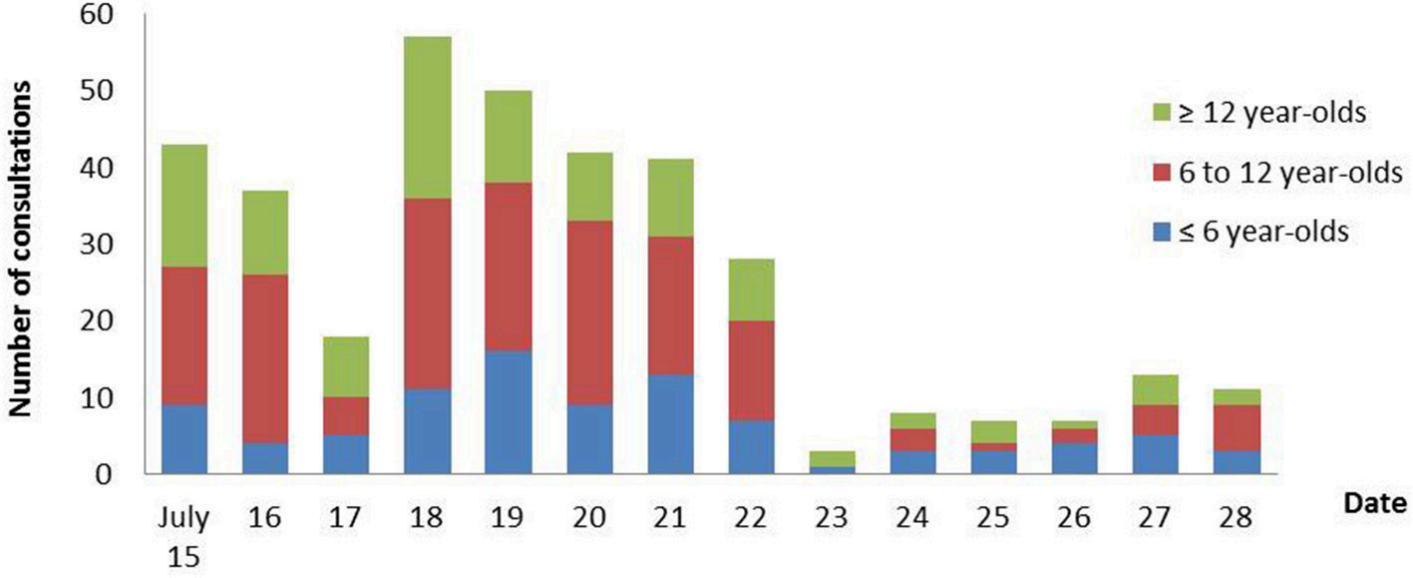
Numbers of consultations for children and adolescents at the Pediatric Medico- Psychological Emergency Cell across the period of July 15–28th, 2016.

**Figure 4:**
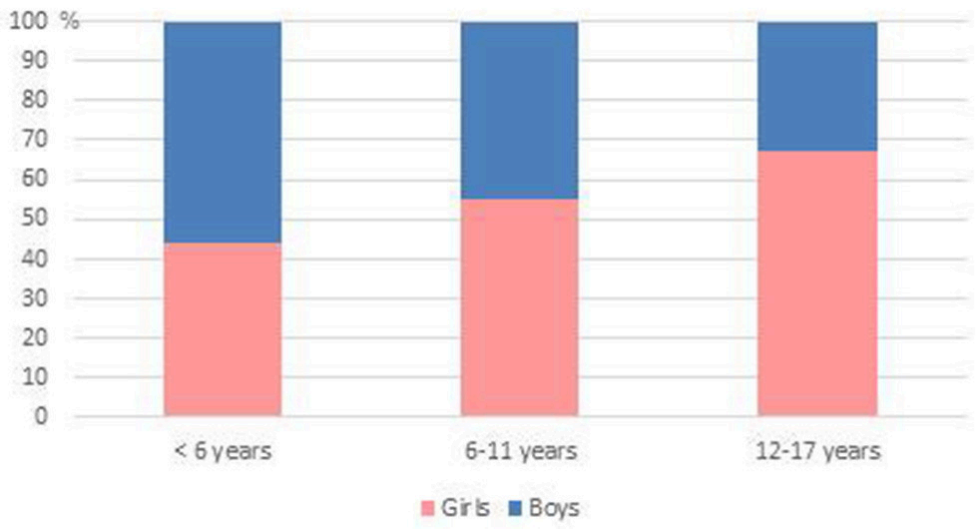
Sex differences according to age. Whereas boys slightly dominated (56 %) before the age of 6, female adolescents clearly constituted the majority of consultations after age of 12.

From July 15–28th, 668 individuals were registered at the pediatric medico-psychological emergency cell, including 365 (54.6%) children and adolescents. Among them, 93 (25.5%) were < 6 years old, 163 (44.7%) were 6 to 11 and 109 (29.8%) 12 to 17 years old. Numbers of consultations for children and adolescents across the period are depicted in Figure 3 with sex differences in Figure 4. Before the age of 6, most consultations concerned boys (*n* = 52, 55.9%) compared to girls (*n* = 41, 44.1%). For children aged between 6 and 11 years, boys represented a slight minority (*n* = 73, 44.8% boys vs. *n* = 90, 55.2% girls). In adolescents aged 12 to 17 years, male adolescents represented only one third of consultations (*n* = 36, 33.0%), the majority having been female adolescents (*n* = 73, 67.0%) (Figure 4).

Mean number of consultations was 70.3 per day during the first eight days, and decreased to 17.7 per day on the following ones. This decrease, together with mentioned staff reinforcements, allowed reducing opening hours from July 22nd. Five Hundred and Seventy-Six (86.3%) of individuals were present on the attack site, 4 (0.6%) had been seriously injured, and 14 (2.1%) were bereaved (with loss of several family members for 4 of them). It can be noted that people came spontaneously to the child and adolescent medico-psychological emergency cell during the first 3 days (July 15–17th), which preceded official announcing and media coverage about the unit.

**Figure 5:**
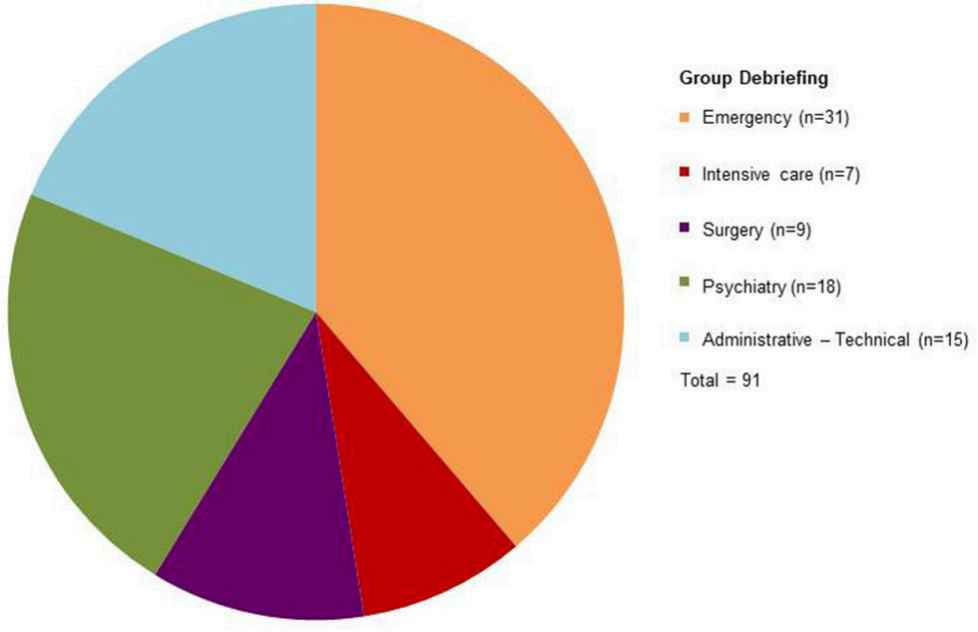
Professionals participating in eight group debriefing sessions.

During the period, somatic polytraumatized children and adolescents and their families were also seen in intensive care and surgery units as part of the liaison psychiatry (22). Here again, mental health professionals were asked to contribute announcing death of a family member or mutilating surgery in some cases, providing thus backup support in such difficult situations and monitoring adverse reactions in children and adolescents. The pediatric medico-psychological emergency cell also provided emotional support to health professionals in all units of the hospital, particularly in the first days following the attack, before the implementation of group debriefing sessions. These group debriefing sessions were instituted for professionals starting from July 17th, 2016, in order to prevent emotional exhaustion and vicarious traumatization. Eight sessions took place over the period, for 91 professionals in total, mainly from emergency (*n* = 31), psychiatry (*n* = 18), administrative-technical (*n* = 15), surgery (*n* = 9), and intensive care (*n* = 7) departments (Figure 5). These sessions were held by psychiatrists and psychologists external to the hospital (from medico-psychological emergency cells of other French departments), together with the medical coordinator of the local child and adolescent medico-psychological emergency cell.

On July 29th, 2016, 2 weeks after the Nice attack, professionals of the national Healthcare Emergencies Preparation and Response Team (*Equipe de Préparation et de Réponse aux Urgences Sanitaires, EPRUS*, see Box 2.5, Figure 1) arrived at the Children's Hospitals of Nice and took over the medico-psychological emergency cell, after information handover with its medical director and health executives.

## Discussion

The purpose of the article was to review child and adolescent psychiatry data regarding the early phase response to the truck-ramming attack of Nice on July 14th, 2016. In this section, we will discuss lessons which could be drawn, with a focus on confronted organizational challenges and applied solutions (see also Table 1).

**Table 1:** Major lessons and recommendations for emergency medico-psychological care for children and adolescents after collective trauma.

**IMMEDIATE RESPONSE PHASE:**
Specific child and adolescent psychiatric component included in all hospitals' disaster plans Quiet location easily accessible close to emergency ward Anticipation of organization, activation but also roles and responsibilities of each professional Dedicated professionals for logistic and organization tasks, not directly involved in patient care Group debriefing of health as well as associated professions (administration, technical staff)
**POST-IMMEDIATE RESPONSE PHASE:**
Specific child, adolescent, and family medico-psychological care Location distant from attack site Organization and supervision by professionals not directly involved in patient care Participation in liaison psychiatry and supporting announcements of severe sequelae or death Systematic group debriefing by external specialists Individual debriefing whenever necessary
**GENERAL:**
Professionals specialized in mental health care for children and adolescents Anticipation of prior training for specificities of post-traumatic emergency care for a very large number of child and adolescent psychiatry professionals Specific evaluation tools for children of all ages Systematic administrative and clinical data collection Structuring mid- and long-term pediatric mental health care in parallel with the post-immediate response
**CLINICAL BUT ALSO RESEARCH PREPAREDNESS:**
Dedicated national and International research networks Clinical research programs for immediate and post-immediate medico-psychological care for children and adolescents Research programs for mid- and long-term follow-up regarding individual, familial and collective outcomes

Regarding the few hours following the event—the immediate response phase—our experience firstly reinforces the importance of specifically providing for child and adolescent aspects within the psychiatric component of hospitals' disaster plans (23). Indeed, clinical interventions for children and adolescent and their families in such context systematically require competences in child development (4, 24), which needs to be anticipated in the case of a general psychiatry facility.

As the main principles of psychological intervention at this stage are the promotion of a relative sense of safety and of calming (9), hospital disaster plans should ideally provide for locating this activity in premises which are both quiet and close to emergency wards and accessible from them. Of course, precise definitions of the mental health staff, the way to activate it, and also of the roles and responsibilities of each professional should be anticipated (23). Moreover, based on our local experience, we consider that logistical and organizational tasks—e.g., layout and equipment of waiting and consultation rooms, general information of patients—should be handled by dedicated professionals—here health executives and the head of the department (20). In addition, special attention should concern the separation of functions and responsibilities, specifically between coordination tasks and patient care.

Most of clinical and organizational issues raised for the immediate response phase are applicable to the following days, the post-immediate response phase. However, a preliminary concern is the opportuneness for the child and adolescent medico-psychological team to stay located close to the pediatric departments, as it was the case here. Nevertheless, the location of the Children's Hospitals close to the attack site may have discouraged some people with acute stress reaction from coming to consultations. However, it might also be conjectured that coming back in this place and being in contact with a fully organized medico-psychological team has allowed a therapeutic early stimulus exposition, and globally promoted a sense of collective effectiveness and hope (9). Another issue is the location of the child and adolescent medico-psychological emergency cell at a distance from the adult ones, which did not always allow a fully integrated functioning of both facilities. It might therefore seem preferable to integrate both facilities, and locate them in a place at a reasonable distance from the attack site. Nevertheless, since the first hours after the disaster, a great number of families immediately and spontaneously converged to the Children's Hospitals of Nice in search of psychological help for acute stress reactions, even sometimes without having been directly exposed to the attack (25). The local organization within the Children's Hospitals also facilitated the implementation of a flexible rotation of child mental health professionals. Special attention is needed in order to prevent vicarious traumatization (26). Here, professionals who were in clinical contact with traumatized individuals worked 1 day per week on average at the child and adolescent medico-psychological emergency cell, and they could be replaced by a colleague when necessary. This required involving a large number of professionals, and could be at best organized within a hospital site including several departments of child and adolescent psychiatry. In addition, the localization permitted direct interventions for in-patient children and families, as well as the direct access to clinical and technical pediatric teams when necessary. However, the close coordination with child as well as adult psychiatry, surgery, emergency and intensive care medicine needs to be anticipated.

It is recommended that post-disaster medico-psychological emergency teams include a medical intervention supervisor, who does not directly interact with patients, and whose specific role is to check upkeep of medical files, validate the list and order of consultations, and detect signs of emotional exhaustion in professionals and giving them breaks when necessary (18, 23). It has indeed been considered that this supervising role should not necessarily be devoted to a physician (23). Another role of the supervisor should be the regulation of the demand of reporters for interviews of patients, and prevent intrusive ones. In the Nice emergency medico-psychological unit, the supervisor functions were dispatched to the medical head of the department health and health executives (8).

Medico-psychological interventions at the emergency cell included a long time period of carefully and empathic hearing as well as therapeutic guidance, regarding for examples management of sleep disturbances and of negative thinking (e.g., shame, guilt, and culpability). They comprised no structured cognitive-behavioral therapeutic intervention. However, this kind of early intervention has shown significant therapeutic effects for acute stress reaction and prevention of PTSD in adults (27). It might thus be considered in the future for children and adolescents and could be implemented and evaluated for selected patients at risk for subsequent PTSD.

More basically, a fundamental element of early psychological intervention for collective trauma is to inform people about the situation and dissipate rumors. Therefore, it is necessary for professionals to have clear and reliable information at their disposal (4, 28). This implies that, in such circumstances, representatives of child and adolescent mental health professionals should be in regular contact with local authorities (23), whose role is also crucial in informing the population about the availability of psychological help for children and adolescents.

Regarding liaison psychiatry, the most significant issue was the demand of the intensive care unit team that mental health professionals actively participated to death notifications. Although such medical teams are regularly confronted with the death of a child or an adolescent and announcing it to parents in ordinary times, the inverse situation of announcing death of one or several of his/her relatives to a child or an adolescent is less frequent. Such demands may also be related to the fact that being exposed to numerous children critically injured in a terrorist attack is an unusual and highly stressing condition for health care workers, which can acutely impair emotional aspects of their professional competences.

In the context of a mass trauma, preventing emotional exhaustion and vicarious traumatization in pediatric health professionals is a major challenge. In France, it includes group debriefing for voluntary professionals (29). It is worth mentioning that it seems preferable that such group debriefings should be held by psychiatrists and psychologists who do not belong to the personnel of the hospital site.

It appears that the main challenge regarding immediate child and adolescent psychiatry response to a mass terrorist attack is the preparedness of professionals, and particularly their prior training in this clinical domain. Indeed, child and adolescent mental health professionals should know how to simultaneously promote safety, calming, and positive emotions, without neither being intrusive nor appearing judgmental. They should also assess the need for a more formal therapeutic intervention and its degree of urgency. Professionals must also be trained to identify factors associated with ulterior psychiatric disorders in children and adolescents after psychological trauma (4, 10, 11). Those factors include a previous history of psychological trauma or mental disorder, or a family with depleted psychosocial or economic resources (30). Specificities of trauma need also to be considered, such as raw intensity of the exposure to the traumatic event, exposure to casualties or human remains, intensity of acute stress reaction (including associated dissociative symptoms) and/or the loss of a relative or friend in the event (31). In addition, parental distress or feelings of culpability should be taken into account (32). On the basis of these items, it would be necessary in the future to devise and validate a structured facility for early clinical triage of children and adolescents.

Finally, as psychiatric response after mass trauma must focus on long term outcomes, another issue is to associate all departments of child and adolescent psychiatry to preparedness, in order that patients who need more formal and intensive therapeutic interventions from the early phase have a rapid access to evidence-based ones.

## Conclusion

The early phase psychiatric response for children and adolescents after the mass trauma of the Nice terrorist attack of July 14th, 2016, has been an exceptional and unpprecedented challenge for mental health care. Our experiences underline the necessity of a specific emergency psychological response for children, adolescents and their families. Further studies are needed in order to evaluate such early phase psychiatric facilities, regarding their input to individual, familial as well as collective outcome. National and international research networks should help to develop clinical but also research preparedness of medico-psychological care facilities for similar emergency situations.

## Author Contributions

FA, FG, and ST coordinated the conceptualization of the study. MG, LC, and ST analyzed the data of the study. FA, FG and ST have been involved in drafting the first version of the manuscript, and all authors have revised it critically for important intellectual content, and approved the final version. All authors were involved in the interpretation of results.

### Conflict of Interest Statement

The authors declare that the research was conducted in the absence of any commercial or financial relationships that could be construed as a potential conflict of interest.
